# Interactions between Plant Metabolites Affect Herbivores: A Study with Pyrrolizidine Alkaloids and Chlorogenic Acid

**DOI:** 10.3389/fpls.2017.00903

**Published:** 2017-05-30

**Authors:** Xiaojie Liu, Klaas Vrieling, Peter G.L. Klinkhamer

**Affiliations:** Plant Ecology and Phytochemistry, Institute of Biology, Leiden UniversityLeiden, Netherlands

**Keywords:** antagonistic interactions, synergistic interactions, plant defense, insect herbivores, predominant storage

## Abstract

The high structural diversity of plant metabolites suggests that interactions among them should be common. We investigated the effects of single metabolites and combinations of plant metabolites on insect herbivores. In particular we studied the interacting effects of pyrrolizidine alkaloid (PAs), and chlorogenic acid (CGA), on a generalist herbivore, *Frankliniella occidentalis.* We studied both the predominantly occurring PA *N*-oxides and the less frequent PA free bases. We found antagonistic effects between CGA and PA free bases on thrips mortality. In contrast PA *N*-oxides showed synergistic interactions with CGA. PA free bases caused a higher thrips mortality than PA *N*-oxides while the reverse was through for PAs in combination with CGA. Our results provide an explanation for the predominate storage of PA *N*-oxides in plants. We propose that antagonistic interactions represent a constraint on the accumulation of plant metabolites, as we found here for *Jacobaea vulgaris*. The results show that the bioactivity of a given metabolite is not merely dependent upon the amount and chemical structure of that metabolite, but also on the co-occurrence metabolites in, e.g., plant cells, tissues and organs. The significance of this study is beyond the concerns of the two specific groups tested here. The current study is one of the few studies so far that experimentally support the general conception that the interactions among plant metabolites are of great importance to plant-environment interactions.

## Introduction

In nature plants are challenged by a multitude of herbivores and pathogens and they protect themselves against these attackers (Hartmann, 2008; Gols, 2014) with an array of defensive strategies, among which the chemical defenses are the most important (Johnson, 2011; Mithofer and Boland, 2012). The plant kingdom has evolved an enormous number (>200,000) of chemically diverse metabolites (Dixon and Strack, 2003; Efferth and Koch, 2011). The chemical diversity is reflected by the wide variety of compounds, including such structurally different groups as glucosinolates, saponins, alkaloids, essential oils, flavonoids, and organic acids (Scott et al., 2002; Mithofer and Boland, 2012). Also within species a large variation in metabolite composition and concentration occurs. For instance, more than 170 specialized metabolites (SMs) (Pichersky and Lewinsohn, 2011) were recorded in *Arabidopsis thaliana* (D’Auria and Gershenzon, 2005). The co-occurrence of plant metabolites within a plant individual provides a high probability for interactions among them. It has become clear that the ability to synthesize SMs evolved in different plant lineages, and these compounds represent adaptations to specific ecological situations, for example, attraction of specific pollinators or defense against specific herbivores. For this reason, we refer to these compounds as “SMs” (Pichersky and Lewinsohn, 2011).

Interactions between plant metabolites can be of great ecological significance with relevance to the following aspects. First, this is in particular of interest for metabolites that are less effective or apparently lack defensive properties on their own (Harborne, 1989; Ayres et al., 1997; Dyer et al., 2003). The contributions of those metabolites to plant defense may be overlooked if they are separated from co-occurring metabolites and are only tested individually. As an example, rutin by itself did not have a negative effect on the growth rate of the caterpillar *Spodoptera exigua*, while CGA had a slight negative effect, but together they had a strong negative effect (Stamp and Yang, 1996). The loss of bioactivity may be caused by the separation of synergistically interacting plant metabolites, which has been found in other research fields, like phyto-medicinal studies, where loss of bioactivity is often reported upon fractionation (Williamson, 2001; Herrera and Amor, 2011; Inui et al., 2012; Labuschagne et al., 2012). In a similar way, interactions may explain why some metabolites show bioactivity in one species while the bioactivity is absent in another. In one species bioactivity comes about through interaction with metabolites that are absent in another. Interactions between metabolites are crucial for a plants defense as plants can benefit from synergism between metabolites if they increase bioactivity at a lower cost (Berenbaum and Zangerl, 1993; Nelson and Kursar, 1999). From an evolutionary point of view, it is therefore expected that natural selection leads to an increased number of synergistic interactions between metabolites to increase overall plant defense. In case a plant produces defense metabolites that are phytotoxic, an antagonistic interaction can be a way to reduce potential self-toxicity but at the same time that would reduce the effectiveness of the defense.

Additionally, interactions between plant metabolites may help to understand the production and/or the storage of weakly active forms of a particular group of metabolites. In this regard, pyrrolizidine alkaloids (PAs) containing species are a good study system because PAs are reported to occur mainly as PA *N*-oxides which in general are less active than the corresponding free bases in fending off insect herbivores (Dreyer et al., 1985; Hartmann et al., 1989; van Dam et al., 1995; Macel et al., 2005; Hartmann, 2007; Nuringtyas et al., 2014; Liu et al., 2017).

In spite of the great significance, the interactions between plant metabolites on plant defense have been scarcely investigated (but see the references below). This is in part due to the complexity of potential interactions between numerous plant metabolites, and to the difficulty of analyzing interactions in a proper manner (Nelson and Kursar, 1999). Regarding the complexity of the interactions, a way forward would be to start with combining two specific metabolites which may interact with each other with a high probability. It is such prior information of individual metabolites that forms a starting point for the current study. Regarding the lack of a theoretical framework to analyze interactions, we developed a statistical model to test simultaneously both for synergistic and antagonistic interactions (Liu et al., 2017).

Previous studies mainly focused on interactions between metabolites within a structural related class of metabolites. Examples include the combination of two amides (Dyer et al., 2003; Richards et al., 2010; Whitehead and Bowers, 2014), the combination of two potato glycoalkaloids (Smith et al., 2001), the combination of two iridoid glycosides (Richards et al., 2012), the combination of two linear furanocoumarins (Diawara et al., 1993; Calcagno et al., 2002), the combination of PA free bases and PA *N*-oxides (Macel et al., 2005; Liu et al., 2017) on the performance of herbivores. Perhaps of greater importance is to study interacting effects between metabolites from different chemical classes on plant defense as the chemical properties of different metabolites might facilitate or counteract each other. For a plant defense metabolite to reach the target site(s) and to be active, a metabolite has to pass through several steps in an insect, such as metabolization, detoxification, sequestration, and secretion, etc. (Berenbaum, 2002; Despres et al., 2007; Wu and Baldwin, 2010). Many of these processes can, to different extents, be influenced by metabolites from other chemical classes. For instance, monoterpenes inhibited cytochrome P450 enzymes involved in α-terthienyl (a terthiophene) degradation, thereby increasing the effect of α-terthienyl on the reduction of relative growth rate of the European corn borer *Ostrinia nubilalis* larvae (Guillet et al., 1998).

In this paper, we studied if interactions among plant metabolites affected the mortality of a generalist insect herbivore. We chose two groups of well characterized SMs, the PA free bases and their corresponding PA *N*-oxides and chlorogenic acid (CGA). PAs are well known for their deterrent and/or toxic effects toward generalist insect herbivores, like the western flower thrips, *Frankliniella occidentalis* Pergande (Thripidae) (Macel et al., 2005; Leiss et al., 2009a; Liu et al., 2017), the beet armyworm (*S. exigua*) (de Boer, 1999; Siciliano et al., 2005; Nuringtyas et al., 2014; Jing et al., 2015), and the tobacco budworm (*Heliothis virescens*) (Cogni and Trigo, 2016). In *Jacobaea vulgaris*, except for the minor PAs with an otonecine base, PAs can exist in two interchangeable forms: free base alkaloids and their *N*-oxides. PAs are reported to occur mainly as PA *N*-oxides in plants (Hartmann et al., 1989) although the jacobine-like PAs can occur up to 50% as free bases in plants (Joosten et al., 2011). Previous studies have shown that, in general, PAs free bases are more active than their corresponding PA *N*-oxides in fending off insect herbivores (Dreyer et al., 1985; Hartmann et al., 1989; van Dam et al., 1995; Macel et al., 2005; Hartmann, 2007; Nuringtyas et al., 2014; Liu et al., 2017).

Chlorogenic acid has a widespread occurrence in the plant kingdom. Phenylpropanoids such as CGA may have a mixed effect on herbivores. Bi et al. (1997) found no effect of CGA on caterpillars of the generalist tobacco budworm *H. virescens* and the specialist tobacco hornworm *Manduca sexta* feeding on tobacco, while CGA was found to be involved in resistance to the apple aphid *Aphis pomi* (Miles and Oertli, 1993). Nuessly et al. (2007) reported that maize plant resistance to corn earworm (*Helicoverpa zea*) was partly due to the presence of CGA. The effects of CGA do not seem consistent across plant species. For instance, CGA concentration in *Chrysanthemum* plants was negatively correlated with the feeding damage of the western flower thrips *F. occidentalis* (Leiss et al., 2009b), while no effect of CGA concentration on western flower thrips was detected in tomato, *Solanum lycopersicum* (Mirnezhad, 2011), carrot *Daucus carota* L. (Leiss et al., 2013), and *J. vulgaris* (Leiss et al., 2009a). These results suggest that the effect of CGA is depending on the chemical background of the different plant species.

Preliminary studies with *S. exigua* cell lines showed antagonistic interactions between jacobine and CGA (Nuringtyas, 2014). In addition PAs and CGA concentration differ between plant cell layers in *J. vulgaris* (Nuringtyas et al., 2012). In the mesophyll of *J. vulgaris* PAs were present in high concentrations and CGA concentration were low while in the epidermis PAs were present in low concentrations and CGA concentration were high (Nuringtyas et al., 2012). It is further known that CGA is able *in situ* to form a complex with caffeine (a purine alkaloid) (Mösli Waldhauser and Baumann, 1996). We therefore hypothesized that PAs and CGA in the diet act antagonistically on the survival of the western flower thrips (*F. occidentalis*). In this study, we tested if interactions between CGA and PAs (free bases and *N*-oxides) affected the mortality of the western flower thrips (*F. occidentalis*).

## Materials and Methods

### Metabolites

**FIGURE 1:**
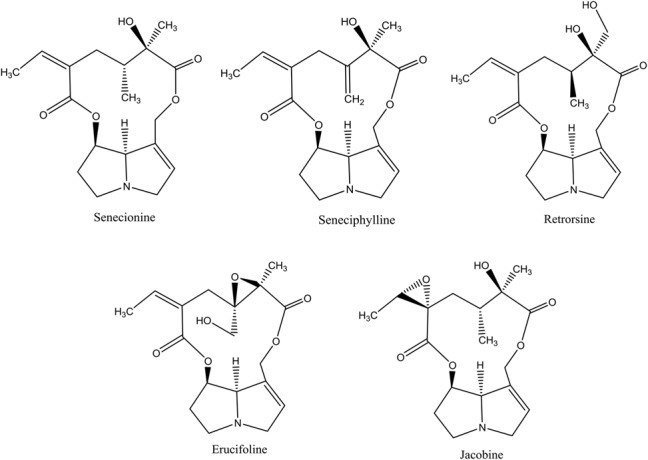
The chemical structures of pyrrolizidine alkaloids (PAs) used in this study. All PAs are depicted as free bases.

More than 37 different PAs have been identified in *J. vulgaris* (Cheng et al., 2011a). We tested five 12-membered macrocyclic PAs and their respective *N*-oxides, which occur in *J. vulgaris*. These PAs represent the three most abundant PA groups within *J. vulgaris*: senecionine-like (senecionine, seneciphylline, and retrorsine), erucifoline-like (erucifoline) and jacobine-like (jacobine) (**Figure 1**), which are divided on the basis of the biosynthetic pathway (Cheng et al., 2011a). An 11-membered macrocyclic PA, monocrotaline, occurring in *Crotalaria* species, was included as well.

#### PA Free Bases

Retrorsine (Sigma, St. Louis, MO, United States), senecionine (Phytoplan Diehm and Neuberger GmbH, Heidelberg, Germany) and monocrotaline (Carl Roth, Karlsruhe, Germany) were purchased. The extraction and isolation of seneciphylline, jacobine, and erucifoline were performed by Explant, Leiden, The Netherlands. They were isolated by centrifugal partition chromatography (CPC; Kromaton FCPC, Annonay, France) from an extract of different chemotypes of *J. vulgaris*. Jacobine was extracted from *J. vulgaris* jacobine chemotypes collected in Meijendel, The Netherlands while erucifoline was extracted from a *J. vulgaris* erucifoline chemotype collected near Filly in Belgium.

Further purification was performed on a Shimadzu preparative High Performance Liquid Chromatography (*p*HPLC; Suzhou, China) equipped with quaternary pumps (LC-10AD), a Rheodyne injection valve with a 20 μL loop, and UV-VIS detector (SPD-10A) connected in a fraction collector (FRC-10A) (Liu et al., 2017). The separation was performed on a C_18_ Luna column (5 μm, 250 mm × 21.20 mm, Phenomenex, Torrance, CA, United States) at 9.9 mL/min of flow, column temperature at 29°C. Afterwards, the four isolated pure PAs were identified by LC-MS by EXPLANT, Leiden, The Netherlands using commercial standards (Liu et al., 2017).

#### PA *N*-Oxides

Retrorsine *N*-oxide (R-0507, Lot 31K1407) was purchased from Sigma Aldrich (St. Louis, MO, United States). The other three *N*-oxides, senecionine *N*-oxide, seneciphylline *N*-oxide and jacobine *N*-oxide were obtained by *N*-oxidation of the corresponding PAs free base by EXPLANT, Leiden, The Netherlands. Briefly, PA free bases were oxidized with *m*-chloroperoxybenzoic acid (*m*-CPBA) (Zalkow et al., 1985) and purified by preparative HPLC using column chromatography with a C_18_ Luna column (5 μm, 250 mm × 21.2 mm, Phenomenex, Torrance, CA, United States). Further purification and identification were performed by EXPLANT, Leiden, The Netherlands (Liu et al., 2017).

**FIGURE 2:**
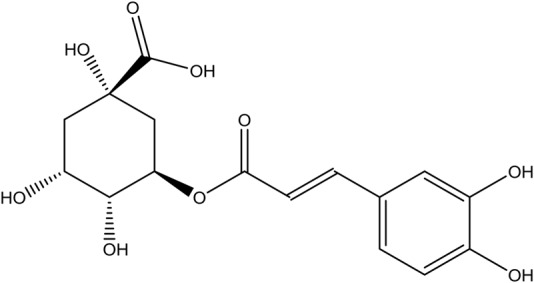
The chemical structure of chlorogenic acid (CGA).

Chlorogenic acid (C-3878) was purchased from Sigma (St. Louis, MO, United States) (**Figure 2**). Abamectin (31732; Sigma, United States) was used as a positive control.

### Thrips Bioassay

Second instar larvae of the western flower thrips, *F. occidentalis* Pergande (Thripidae), were obtained from a lab culture reared on chrysanthemum flowers in a growth chamber at 23°C, 12h L/D photoperiod, 60% RH. Western flower thrips bioassays were conducted in adapted 96-well plates filled with 55 μL test solutions covered with parafilm (Liu et al., 2017). Single second instar larvae of thrips were placed into each lid of an 8 cup flat-cup strip. Each lid was sealed with parafilm and then on top of the 96 well plates. The 96 wells plates were placed upside down so that thrips were able to feed from the offered test solutions by piercing through the parafilm. The plates were randomly placed into a growth chamber with standard thrips rearing conditions (L:D, 12:12, 23°C).

The test solution consisted of 10% fructose and a phosphate buffer (NaH_2_PO_4_ and Na_2_HPO_4_, 40 mM, pH 7). PAs were dissolved in MeOH and added to this solvent so that the final concentration of MeOH was always 3%. The negative control consisted of a 3% MeOH in the test solution. There were two positive controls for each group: empty wells (without solution) to verify that western flower thrips larvae could not survive without feeding and a solution with the insecticide abamectin (50 μg/mL) to have a positive control to compare it to the effects of PAs.

One replicate consisted of a bioassay carried out in eight 96-well plates. Per two 96-well plates there are 24 columns (1-24) of 8 wells. A column of 8 wells received the same treatment. Of the 24 columns, 18 columns were filled with 18 different treatments and 6 columns were left empty. The 18 treatments included the negative control (only the solvent), 15 treatment groups [i.e., 3 concentrations of a PA, 4 concentrations of CGA and 8 concentrations of combinations of PAs and CGA] and two positive controls (empty wells and abamectin). As in total there were eight 96 wells plates each treatment therefore consisted of 32 wells. Each bioassay was repeated on different days yielding two independent replicates for all treatments (two replicates with 32 thrips larvae). By doing so, two independent estimates of thrips survival (*n* = 2) are obtained for the analysis of variance. After 5 days, the numbers of surviving larvae were counted with a stereo microscope. Mortality was calculated as the number of dead larvae in a treatment in one replicate divided by the total number of larvae (32) in that treatment. All replicates had their own negative control and the correction of thrips survival was therefore performed per replicate.

### Experiments of Individual PAs in Combination with CGA

Three doses of 0, 4 and 7 mM of retrorsine, jacobine, erucifoline and monocrotaline, respectively, were combined with five concentrations of 0, 1.4, 2.8, 5.6, and 14 mM CGA yielding 15 combinations including the tests with single metabolites. Due to its limited solubility in the test solution, senecionine was only tested at concentrations of 0, 0.8 and 1.4 mM.

In a similar way, three doses of 0, 4 and 7 mM of senecionine *N*-oxide, seneciphylline *N*-oxide, retrorsine *N*-oxide and jacobine *N*-oxide, respectively, were combined with five concentrations of 0, 1.4, 2.8, 5.6, and 14 mM of CGA.

One PA, retrorsine, was selected for further investigation because it provided the strongest interaction with CGA and the statistical analysis showed that the magnitude of the interaction depended also on the concentration of CGA. We therefore tested a wider range of 30 combinations of retrorsine with CGA (Supplementary Figure S1).

### Statistical Analysis

#### Construction of an Interaction Model

To evaluate synergistic and antagonistic effect of the interactions between two metabolites, we developed a statistical model (Liu et al., 2017). We first constructed a “null interaction” model that predicts the effect of metabolites in the absence of interaction. A multiplicative null model was constructed, which is based on the idea of probabilistic independence of the effect of combinations of drugs (Bliss, 1939; Berenbaum, 1989).

In a multiplicative null model, assuming no interaction effects, the effect of the combination is the product of the effects of the two metabolites, i.e., *S*_X+Y_ = *S*_X_ ∗ *S*_Y_. The underlying assumption of the multiplicative model is that the relationship between log-transformed survival of thrips larvae against the concentration of the tested individual metabolites is linear.

Thrips survival data was therefore log-transformed to obtain linear relationships with the concentrations of CGA and retrorsine. To determine whether there was a linear dose-related decrease, log-transformed survival was regressed against concentrations (see results in Supplementary Figure S2).

Based on the regression models, the effect of the combination of two metabolites in the absence of interaction was calculated.

#### Correcting for Survival in the Negative Control

The survival in the negative control was in all cases high (>86%). Nevertheless, we corrected survival data for differences among the negative controls in the following way.

The observed experimental survival of larvae (*S*_X+NC_) results from the survival after application of tested metabolite (*S*_X_) and from the survival in the solvent (*S*_NC_). The survival of the solvent is measured in the negative control. Under the assumption that the two effects are independent, we get the following equation:

(1) $$\begin{array}{c} S_{X}{{}_{+}}_{\mathrm{NC}}\;=\;S_{\mathrm{NC}}*S_{X} \end{array}$$

The survival resulting from the application of the metabolite S_X_ can thus be calculated as

(2) $$\begin{array}{c} S_{X}\;=\;S_{X}{{}_{+}}_{\mathrm{NC}}/S_{\mathrm{NC}} \end{array}$$

#### Testing the Interaction Effects of the Combination of Two Metabolites on Thrips

In the case of a combination, the survival of thrips for the combination of metabolites X with Y (*S*_X+Y_) results from the survival after application of the single metabolite X (*S*_X_), the survival after application of the single metabolite Y (*S*_Y_), and their interaction (*S*_X∗Y_).

(3) $$\begin{array}{c} S_{X}{{}_{+}}_{Y}\;=\;S_{X}*S_{Y}*S_{X*Y} \end{array}$$

Therefore, the effect of the interaction of two metabolites can be calculated by:

(4) $$\begin{array}{c} S_{X*Y}\;=\;S_{X}{{}_{+}}_{Y}/(S_{X}*S_{Y}) \end{array}$$

In which *S*_X+Y,_ *S*_X_, and *S*_Y_ are derived using equation (2). In equation (4), *S*_X+Y_ is the observed survival in experiments with combinations while *S*_X_ and *S*_Y_ are the observed thrips survival in experiments with single metabolites while *S*_X∗Y_ denotes the interaction effect. Note that the interaction effect thus denotes the effect of the interaction between the PA and CGA on the survival of thrips while 1-*S*_X∗Y_ denotes the fraction mortality caused by this interaction.

As each experiment is performed twice, two independent estimates of survival are obtained from which we can calculate *S*_X∗Y_ (*n* = 2). To estimate if the interaction effect *S*_X∗Y_ deviates significantly from one, a two-way analysis of variances (ANOVAs) were used with the concentration of metabolites X and Y as factors. As dependent variables the two estimates of *S*_X∗Y_-1 = [*S*_X+Y_ / (*S*_X_ ∗*S*_Y_)]-1 were used for all combinations of traits. Therefore, if the intercept of two-way ANOVA is significantly deviating from zero it indicates that *S*_X∗Y_-1 is deviating from zero and hence that *S*_X∗Y_ is significantly deviating from one. If the interaction effect *S*_X∗Y_ is > 1 it indicates an antagonistic interaction and if it is < 1 it indicates a synergistic interaction. To avoid confusion with statistical interaction terms of ANOVA and multiple regression we will always refer to the value of the interaction effect between the metabolites as the “interaction effect *S*_X∗Y._”

The strengths of the antagonistic effects were compared by three-way ANOVAs with PA, PA concentration and CGA concentration as fixed factors and the interaction effect *S*_X∗Y_ as the dependent variable (*n* = 2). As senecionine was tested at different concentrations than the other four PAs, it was not included in the three-way ANOVA.

To display the effects of the single metabolites and their combinations on thrips, we plotted fraction mortality (i.e., 1 – fraction survival) against the concentrations of the two metabolites in a 3-dimensionsial (3D) graph with the 2 horizontal axes (*x* and *y*) representing the two metabolite concentrations, and the vertical axis (*z*) representing thrips mortality.

To visually examine the most effective combination, a heat-map of the interaction effects *S*_X∗Y_ from 30 combinations of retrorsine free base with CGA were plotted, with CGA concentration on the *x* axis and retrorsine concentration on the *y* axis (Supplementary Figure S3). Interaction effects *S*_X∗Y_ were also plotted against the log-transformed ratios of retrorsine free base and CGA.

#### Comparison of the Interaction Effects of PA Free Bases and the Corresponding *N*-Oxides with CGA

To compare the interaction effects of PA free bases and PA *N*-oxides combined with CGA on thrips survival, we plotted CGA concentration against the value of Δ thrips survival. Δ thrips survival is the average survival of the combination of a particular PA free base concentration and particular CGA concentration minus the average survival of the corresponding combination of the PA *N*-oxide and CGA concentration. The standard deviation (σ) of the Δ thrips survival was calculated by equation (5), where σ (PA *N*-oxide) and σ (PA free base) are the standard deviation of the thrips survival from the experiment with the PA *N*-oxide combined with CGA and the standard deviation of the thrips survival from the experiment with the PA free base combined with CGA.

(5) $$\begin{array}{c} \sigma \;(\Delta \mathrm{thrips}\mathrm{survival})\;=\;\sqrt{\;\sigma \;{\left(\mathrm{PA}\;N-oxide\right)}^{2}\;+\;\sigma \;{\left(\mathrm{PA}\mathrm{free}\mathrm{base}\right)}^{2}} \end{array}$$

The 95% confidence intervals (CIs) of Δ survival were then estimated by equation (6).

(6) $$\begin{array}{c} 95\%\mathrm{CIs}\;=\;\frac{\sigma \;(\Delta \;thripssurvival)}{\sqrt{n}}*\;1.96 \end{array}$$

With σ (Δ thrips survival) being the standard deviation from equation (5) and *n* being number of measurements for each combination group (*n* = 2). 95% CIs are used to determine if Δ thrips survival is significantly deviating from zero. A Δ thrips survival > 0 indicates that the average thrips survival is lower in the presence of the PA *N*-oxides and a Δ thrips survival < 0 indicates that thrips survival is lower in the presence of the PA free bases.

All statistical analysis were performed using SPSS software for Windows (version 21.0; SPSS Inc., Chicago, IL, United States).

## Results

Western flower thrips survival in the negative control was in all cases higher than 86%. The positive control with the insecticide abamectin (50 μg/mL) showed an average survival of 6.6%. The control with empty wells had an average survival of 4.5% showing that thrips cannot survive without eating.

### Antagonistic Interaction Effects between PA Free Bases and CGA on Thrips Mortality

**FIGURE 3:**
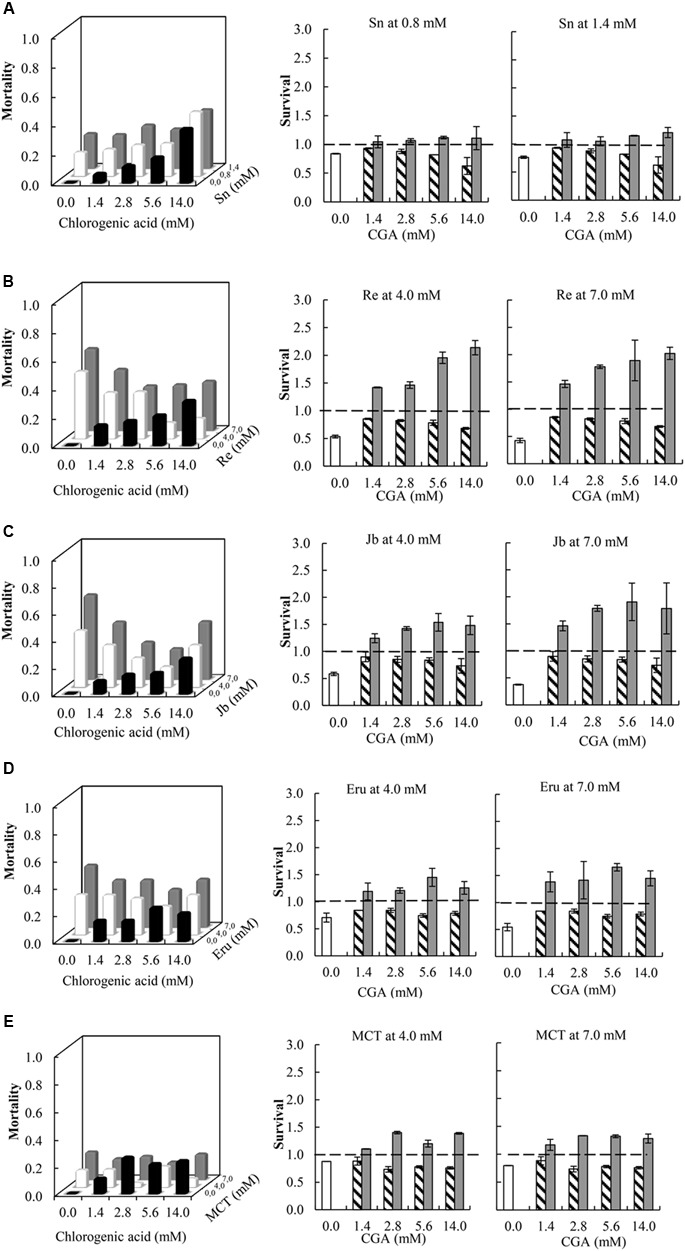
**Left:** Fraction mortality (1- fraction survival) of 2nd instar thrips larvae (*Frankliniella occidentalis*) caused by the single metabolites and the combination of CGA with senecionine **(A)**, retrorsine **(B)**, jacobine **(C)**, erucifoline **(D)**, and monocrotaline **(E)**. **Right:** Fraction survival (mean ± 95% confidence intervals) of thrips caused by PA alone (white bars), CGA alone at four concentrations (hatched bars), and the interaction effect *S*_X∗Y_ (Equation 4) between PAs and CGA (gray bars). In the left figures, the fraction mortality was plotted to increase the readability of the figure. In the right figures, dashed line indicates a thrips survival fraction of one. Deviation of the interaction effect *S*_X∗Y_ from the dashed line (survival = 1) indicates synergistic (<1) or antagonistic effect (>1). Two-way ANOVAs were used to analyze whether the overall interaction effect *S*_X∗Y_ deviated from one (**Table 1**). Sn, senecionine; Re, retrorsine; Jb, jacobine; Eru, erucifoline; MCT, monocrotaline.

Compared to single metabolites, the combinations of all PA free bases with CGA showed a decreased thrips mortality (**Figure 3** and **Table 1**; note that the fraction mortality was plotted in the 3D figures in order to increase the readability of the figure). For retrorsine and monocrotaline the main effect of CGA concentration on the interaction effect S_X∗Y_ is significant indicating that for these two PAs the strength of the antagonistic interaction is dependent on CGA concentration (**Table 1**).

**Table 1:** Two-way ANOVAs with PA free bases concentration and chlorogenic acid (CGA) concentration as fixed factors and the interaction effect minus one (*S*_X∗Y_^-1^) as the dependent variable.

Factors	*df*	*F*	*P*
Intercept	1, 15	6.979	<0.05
CGA conc.	3, 15	0.285	NS
Senecionine conc.	1, 15	0.226	NS
CGA conc. ^∗^Senecionine conc.	3, 15	0.177	NS
Intercept	1, 15	450.121	<0.001
CGA conc.	3, 15	10.270	<0.01
Retrorsine conc.	1, 15	1.797	NS
CGA conc. ^∗^Retrorsine conc.	3, 15	2.807	NS
Intercept	1, 15	87.780	<0.001
CGA conc.	3, 15	1.566	NS
Jacobine conc.	1, 15	1.411	NS
CGA conc. ^∗^Jacobine conc.	3, 15	0.055	NS
Intercept	1, 15	48.814	<0.001
CGA conc.	3, 15	1.164	NS
Erucifoline conc.	1, 15	1.231	NS
CGA conc. ^∗^Erucifoline conc.	3, 15	0.011	NS
Intercept	1, 15	244.857	<0.001
CGA conc.	3, 15	6.964	<0.05
Monocrotaline conc.	1, 15	0.276	NS
CGA conc. ^∗^Monocrotaline conc.	3, 15	2.595	NS

*Survival was corrected for differences among the negative controls (see M&M). A significant intercept indicates a synergistic or antagonistic interaction because if *S*_X∗Y_ = 1 there is no interaction effect. NS = Not significant.*

**FIGURE 4:**
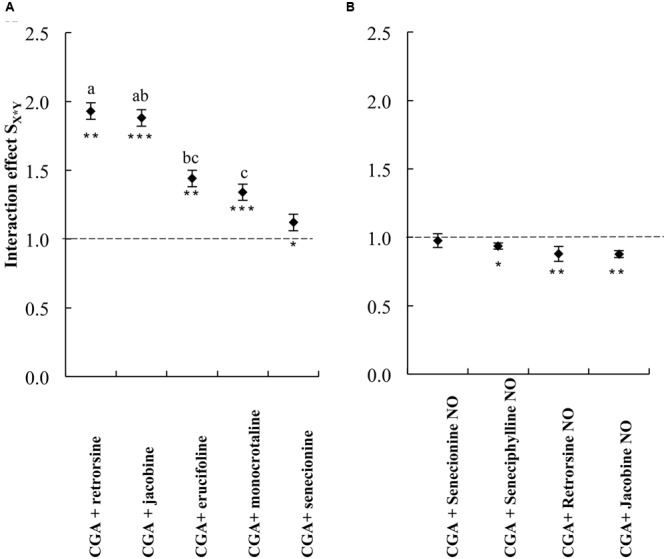
The interaction effect *S*_X∗Y_ on thrips (*F. occidentalis*) larval survival (mean ± S.E.) of the combinations of CGA with five PA free bases **(A)** and PA *N*-oxides **(B)**. Data are from two independent bioassays. Deviation of the interaction effect *S*_X∗Y_ from the dashed line indicates synergistic (<1) or antagonistic effect (>1). The interaction effects *S*_X∗Y_ were analyzed by a three-way ANOVA with PA, PA concentration and CGA concentration as factors (**Table 2**). Different letters indicate that the interaction effects with CGA are significantly different among PAs (Tukey *post hoc* test). Senecionine was excluded from the three-way ANOVAs as it was not tested at higher concentrations. ^∗^*P* < 0.05, ^∗∗^*P* < 0.01, and ^∗∗∗^*P* < 0.001. NO, *N*-oxide.

We then compared the strength of the interaction effects S_X∗Y_ for different PAs, except senecionine, in a three way ANOVA followed by a *post hoc* test. Across all PA free bases we found that the combination of PAs with CGA decreased thrips mortality compared to the effects of the single compounds. The strength of the antagonistic effect significantly differed between retrorsine and erucifoline/monocrotaline, and between jacobine and monocrotaline (**Figure 4** and **Table 2**). The ranking of the mean interaction effect S_X∗Y_ (± S.E.) starting with the highest was as follows: CGA + retrorsine (1.93 ± 0.06) > CGA + jacobine (1.88 ± 0.06) > CGA + erucifoline (1.44 ± 0.06) > CGA + monocrotaline (1.34 ± 0.06) > CGA + senecionine (1.12 ± 0.06) (**Figure 4A** and **Table 2**).

**Table 2:** Three-way ANOVA with PA free base, CGA concentration and PA concentration as factors and the interaction effect *S*_X∗Y_ as a dependent variable.

Factors	*df*	*F*	*P*
Intercept	1, 63	2531.6	<0.001
PA	3, 63	13.4	<0.001
CGA conc.	3, 63	5.8	<0.01
PA concentration	1, 63	5.5	<0.05
PA ^∗^ CGA conc.	9, 63	1.2	NS
PA ^∗^ PA conc.	3, 63	1.5	NS
CGA conc. ^∗^ PA conc.	3, 63	0.2	NS
PA ^∗^ CGA conc. ^∗^ PA conc.	9, 63	0.2	NS

The PA free bases are retrorsine, jacobine, erucifoline, and monocrotaline. Survival was corrected for differences among the negative controls (see M&M). Senecionine was excluded from the three-way ANOVAs as it was not tested at the higher concentrations. NS = Not significant.

In the case of retrorsine and monocrotaline, there is a valley in the 3D plot of thrips mortality (**Figures 3B,E**). The antagonistic effect increases with CGA concentration but becomes smaller as the CGA concentration exceeds 2.8 mM. The dependence of the antagonistic interaction on CGA and retrorsine concentrations was explored further by testing 30 combinations of retrorsine with CGA concentrations (Supplementary Figure S3). Interaction effects largely depended on the concentration of retrorsine (*Y* axis in the heat-map) and the ratio of the concentrations (as indicated by the valley in the heat-map), but less on the concentration of CGA (*X* axis in the heat-map).

### Synergistic Interactions Effects between PA *N*-Oxides and CGA on Thrips Mortality

**FIGURE 5:**
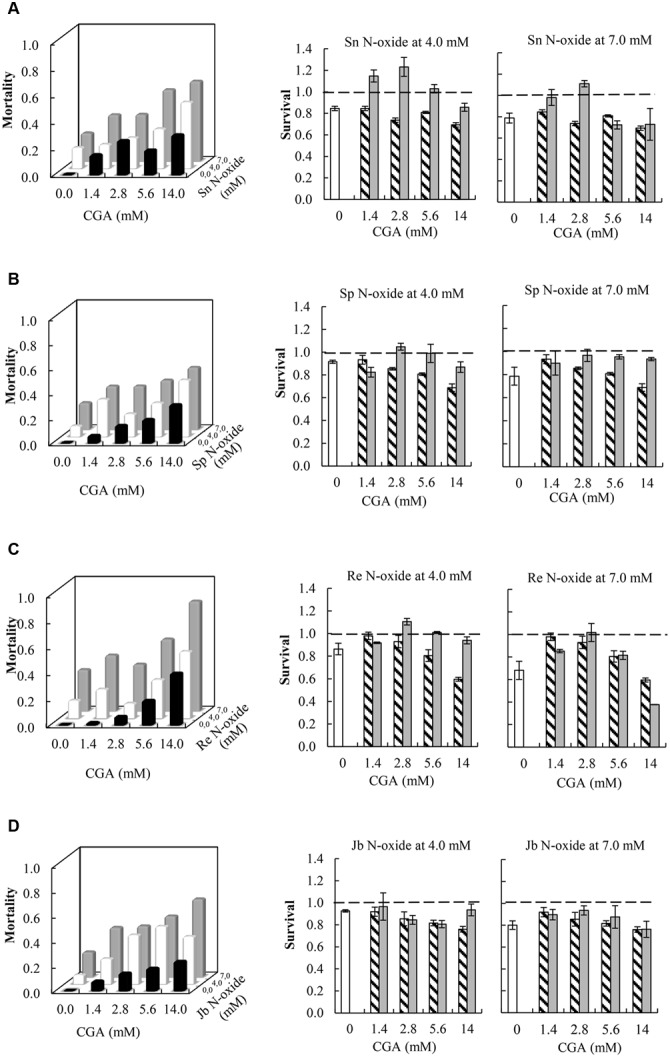
**Left:** Fraction mortality (1- fraction survival) of 2nd instar thrips larvae (*F. occidentalis*) caused by single metabolites and the combination of CGA with senecionine *N*-oxide **(A)**, seneciphylline *N*-oxide **(B)**, retrorsine *N*-oxide **(C)**, and jacobine *N*-oxide **(D)**. **Right:** Fraction survival (mean ± 95% confidence intervals) of thrips caused by PA *N*-oxides alone (white bars), CGA alone at four concentrations (hatched bars), and the interaction effect *S*_X∗Y_ (Equation 4) between PA *N*-oxides and CGA (gray bars). In the left figures, the fraction mortality was plotted to increase the readability of the figure. In the right figures, the dashed line indicates a thrips survival fraction of one. A significant deviation from one indicates a synergistic (<1) or antagonistic effect (>1). Two-way ANOVAs were used to analyze whether the overall interaction effect S_X∗Y_ deviated significantly from one (**Table 3**). Sn *N*-oxide, senecionine *N*-oxide; Sp *N*-oxide, seneciphylline; Re *N*-oxide, retrorsine *N*-oxide; Jb *N*-oxide, jacobine *N*-oxide.

All the combinations of PA *N*-oxides with CGA decreased thrips survival, indicating synergistic interactions (**Figure 5** and **Table 3**). All significant interaction effects, *S*_X∗Y_, were < 1 indicating synergistic effects except for senecionine *N*-oxide (**Figure 5** and **Table 3**). The interaction effects between the four PA *N*-oxides and CGA were not significantly different (**Figure 4B** and **Table 3**). For senecionine *N*-oxide, both main effects, senecionine *N*-oxide and CGA concentration, were significant although the intercept was not significant. The interaction effect *S*_X∗Y_ showed synergistic interactions at the highest concentration while antagonistic effects were present at the lowest concentrations (**Figure 5A**).

**Table 3:** Two-way ANOVAs with PA *N*-oxide concentration and CGA concentration as fixed factors and the interaction effect (Equation 4) minus one (S_X∗Y_-1) as a dependent variable.

Factors	*df*	*F*	*P*
Intercept	1, 15	0.8	NS
CGA concentration	3, 15	10.4	<0.01
Senecionine *N*-oxide conc.	1, 15	11.4	<0.01
CGA conc. ^∗^ Senecionine *N*-oxide conc.	3, 15	0.6	NS
Intercept	1, 15	6.2	<0.05
CGA conc.	3, 15	1.4	NS
Seneciphylline *N*-oxide conc.	1, 15	0.2	NS
CGA conc. ^∗^ Seneciphylline *N*-oxide conc.	3, 15	0.6	NS
Intercept	1, 15	90.1	<0.001
CGA conc.	3, 15	42.7	<0.001
Retrorsine *N*-oxide conc.	1, 15	80.0	<0.001
CGA conc. ^∗^ Retrorsine *N*-oxide conc.	3, 15	20.5	<0.001
Intercept	1, 15	93.3	<0.001
CGA concentration	3, 15	2.2	NS
Jacobine *N*-oxide conc.	1, 15	1.2	NS
CGA conc. ^∗^ Jacobine *N*-oxide conc.	3, 15	4.1	<0.05

*Survival was corrected for differences among the negative controls. A significant intercept indicates that synergistic or antagonistic interactions occur. For all significant intercepts, the interaction effects *S*_X∗Y_ were smaller than one indicating synergistic interactions (see also **Figure 5**). NS = Not significant.*

For seneciphylline *N*-oxide, the strength of the synergistic effect was independent from the concentrations of the two metabolites. In the case of retrorsine *N*-oxide the strength of the synergistic effect also depended on the concentrations of both metabolites and the concentrations at which they were combined (**Figure 5C** and **Table 3**). For jacobine *N*-oxide, the strength of the synergistic effect did not depend on the concentrations of the two metabolites while it did depend on the combinations of the concentrations (**Figure 5D** and **Table 3**).

The interaction effects *S*_X∗Y_ are relatively small and vary between 0.7 and 1.1 (**Figure 5**) except for the combination of retrorsine *N*-oxide at 7 mM with CGA at 14 mM. For all PA *N*-oxides only two out of 16 combinations showed interaction effects *S*_X∗Y_ < 1, indicating that mostly synergistic interactions between the PA *N*-oxides and CGA occur. The strongest interaction effect was found for the interaction between retrorsine *N*-oxide at 7 mM and CGA at 14 mM (*S*_X∗Y_ = 0.37 ± 0.001). The survival of retrorsine *N*-oxide alone was 0.68 ± 0.08 and that of CGA alone was 0.60 ± 0.02 (**Figure 5C**). This means that the expected survival if there is no interaction is 0.68 ^∗^ 0.60 = 0.41. The observed survival was much lower (*S*_X∗Y_ = 0.15) than the expected survival (0.41). From this, it follows that the interaction effect (*S*_X∗Y_) was 0.15/0.41 = 0.37.

### Comparison of the Interaction Effects between the PA Free Bases and PA *N*-Oxides with CGA

**FIGURE 6:**
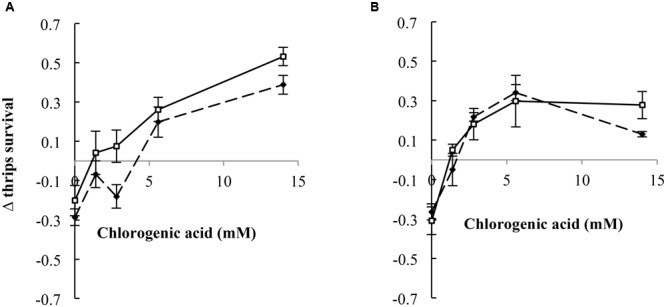
Δ thrips survival (thrips survival for the combination of free base PAs with CGA minus thrips survival for the combination of PA *N*-oxides with CGA (± 95% confidence interval) against CGA concentration. **(A)** Retrorsine, **(B)** jacobine. Dashed lines: PA concentration of 4 mM, solid lines: PA concentration of 7 mM.

We compared thrips survival from these two experiments for retrorsine and jacobine by calculating the difference in thrips survival between the two experiments, the Δ thrips survival. In the case of jacobine and retrorsine, the PA free bases alone resulted in a negative Δ thrips survival indicating a lower thrips survival on free bases compared to the PA *N*-oxides (**Figure 6**). Generally, we found that the Δ thrips survival for the combination between PA *N*-oxides with CGA are positive indicating a lower thrips survival for the combinations of PA *N*-oxides with CGA compared to the combination of PA free bases with CGA. For retrorsine, Δ thrips survival depends on the CGA concentration (**Figure 6A**). The higher the CGA concentration, the lower the thrips survival of retrorsine and jacobine *N*-oxide and CGA in comparison with that of free base retrorsine and jacobine and CGA (**Figure 3**).

## Discussion

The data support the hypothesis that PA free bases and CGA act antagonistically on Western flower thrips mortality which is in agreement with preliminary results of Nuringtyas (2014). In contrast, PA *N*-oxides showed synergistic interactions with CGA on thrips mortality. We subsequently contrasted the effect of retrorsine and jacobine free bases and their *N*-oxides on thrips survival in combination with increasing CGA concentrations. In the absence of CGA, PA free bases had stronger negative effects on thrips survival than that of PA *N*-oxides underpinning the previous findings of the bioactivity of individual PAs and PA *N*-oxides (Dreyer et al., 1985; Hartmann et al., 1989; van Dam et al., 1995; Macel et al., 2005; Hartmann, 2007; Nuringtyas et al., 2014; Liu et al., 2017). However, this effect was reversed when CGA was added showing the significant importance of interactions between different plant metabolites. This study shows that interactions between plant metabolites are important. Our study provides additional and novel information on the bioactivity of metabolites. In particular it shows that the effect of metabolites in concert with other metabolites may strongly differ from that as a single compound. This is particularly meaningful for metabolites that are weakly active or inactive alone as they may potentially contribute to plant defense by interacting synergistically with other metabolites.

In the case of PAs, the current findings provide novel information for our understanding of the roles of PAs in plant defense demonstrating both antagonistic interactions between PA free bases and CGA and synergistic interactions between PA *N*-oxides and CGA. The importance of interactions of PAs in plant defense against herbivores has so far been tested for the effects of interactions among PAs only. Macel et al. (2005) found a weak synergistic effect of a mixture of the free bases of senecionine, seneciphylline and senkirkine on *S. exigua* and of a mixture of the free bases of senecionine and seneciphylline on *L. migratoria*. Yet, Siciliano et al. (2005) did not find any synergistic effects of two PAs from *Anchusa strigosa* on *S. exigua*. These two studies have only tested the interaction effects among PA free bases. We previously tested the combinations of the most abundant PA *N*-oxides of *J. vulgaris*, senecionine *N*-oxide, jacobine *N*-oxide and erucifoline *N*-oxide (Joosten et al., 2011). However, we did not find synergistic or antagonistic effects on thrips mortality (Liu et al., 2017).

Previously, it was reported that PA *N*-oxides are less bioactive than their corresponding free bases in warding off insect herbivores (Dreyer et al., 1985; van Dam et al., 1995; Macel et al., 2005; Nuringtyas et al., 2014; Liu et al., 2017). This raised the question why PAs are mostly present in plants in their least active form. A physiological explanation is that *N*-oxides are more soluble and can therefore be better stored and transported (von Borstel and Hartmann, 1986; Hartmann and Toppel, 1987; Lindigkeit et al., 1997). The current findings supply an alternative explanation: in combination with CGA, PA *N*-oxides show synergistic effects on thrips mortality while PA free bases show antagonistic effects on thrips mortality.

The interactions between plant metabolites in this study also underline the point that the bioactivities of an individual metabolites may vary depending on the specific phytochemical contexts. In the case of PAs, synergism between PA *N*-oxides and other SMs could explain some inconsistent results from correlative studies and bioassays on individual PA *N*-oxides. In a correlative study, Cheng et al. (2011b) found that thrips damage on *Jacobaea* plants decreased with increasing concentration of jacobine *N*-oxide. In contrast, we did not find a dose-dependent effect of jacobine *N*-oxide on thrips survival in a bioassay with single compounds (Liu et al., 2017). Here we found a significantly negative dose-dependent effect of jacobine *N*-oxide on thrips in combination with CGA. Together with other plant metabolites, PA *N*-oxide may become active resulting in a greater effect than the added up effects of the single metabolites. Such effects are not detected or overlooked by testing single metabolites.

Interactions between plant metabolites could also explain the absence activity of some metabolites in one species while they do show activity in other species. This is the case for CGA (Leiss et al., 2009b; Mirnezhad, 2011).

Given the large amount of plant metabolites, antagonistic interactions are likely to occur. Currently, we know of very few studies that have reported antagonistic interactions (but see Diawara et al., 1993; Nelson and Kursar, 1999; Nuringtyas, 2014). Here, for all five tested PA free bases combined with CGA, significant antagonistic interactions were found on thrips mortality. The current results confirmed the preliminary results of Nuringtyas (2014) who injected larvae of *S. exigua* with a combination of CGA with PA free bases also found antagonistic effects on mortality, showing that such antagonistic effects are not specific to thrips. Antagonistic interactions are not an advantage to plant fitness and are not easily explained from an evolutionary perspective. A solution to avoid or minimize antagonistic effects is compartmentalization. Plants may manage the interactions between metabolites by accumulating metabolites in different organs, tissues, cells and even cell compartments (Nuringtyas et al., 2012; Moussaieff et al., 2013; Kuhlisch and Pohnert, 2015). Such a compartmentalization can be used to keep antagonistically interacting metabolites apart but may also promote synergistic interactions between plant metabolites by storing them in the same compartment. Antagonism therefore may represent a constraint or a trade-off on the accumulation of metabolites (Nelson and Kursar, 1999). Metabolites that interact antagonistically on, e.g., thrips mortality may have benefits for other functions. In the specific case we address here, CGA or PAs may have another positive (and perhaps indispensable) function not related to defense against thrips. The reduction in a specific defense (PA free bases) is largely compensated by the benefits to other functions of PA *N*-oxides.

It would be fruitful to understand the underlying mechanisms of interactions between metabolites. To be bioactive, individual metabolites have to pass several steps of the insects’ defensive system (Berenbaum, 2002; Despres et al., 2007; Wu and Baldwin, 2010). All these steps can be supported or influenced by other metabolites, resulting in interaction effects. For insect herbivores, the underlying mechanisms of synergistic or antagonistic interactions between metabolites are not well understood. Findings from other research fields, e.g., pharmacology, show that an interaction may occur in the kinetic phase (i.e., processes of uptake, distribution, metabolism and excretion) and/or in the dynamic phase (i.e., effects on the receptor, cellular target or organ) (Gee et al., 1996; Williamson, 2001; Zimmermann et al., 2007; Biavatti, 2009; Efferth and Koch, 2011; Labuschagne et al., 2012).

Modification of functional groups, addition/elimination of specific groups, or changing the substitution pattern can be used for in-depth investigation of the involvement of the metabolites in the interactions. The facts that all the tested PA free bases interacted antagonistically with CGA and that PA *N*-oxides interacted synergistically with CGA on thrips mortality suggest that the necine part of the PA molecule is involved in the interaction. In the case of both free bases and *N*-oxides, the structure that all tested PAs have in common is the retronecine or retronecine *N*-oxide base. The macrocyclic ring differs between all PAs. With respect to the CGA molecule, it has been reported that caffeine and CGA *in situ* formed a complex at a ratio of 1:1 (Sondheimer et al., 1961; Horman and Viani, 1972; Chapman and Miller, 1974). If similar complexes would be formed between PAs and CGA, we expect that the observed interaction effects will depend on the ratio of two components. However, we found little evidence that a 1:1 ratio is present in the antagonistic interaction between CGA and PA free bases (Supplementary Figure S3).

Structurally, CGA is the ester of caffeic acid and quinic acid. Further tests are needed to determine the involvement of the CGA molecule in the interaction with PAs. This can be achieved by testing the effects of combinations of PA with derivatives of CGA, quinic acid and caffeic acid.

## Conclusion

The interactions between plant metabolites can have major effects on the defense function of the bioactivity of individual metabolites. Therefore, in ecological studies the bioactivity of individual metabolites should be tested in their natural backgrounds of other metabolites, e.g., adding specific metabolites to plant extracts or fractions of extracts.

## Author Contributions

KV and PK conceived and designed the experiments. XL performed the experiments and analyzed the data and wrote the first draft of the manuscript. All authors contributed substantially to revisions. All authors gave final approval for publication.

## Conflict of Interest Statement

The authors declare that the research was conducted in the absence of any commercial or financial relationships that could be construed as a potential conflict of interest.
