# Supplementary material for: Interactions between Plant Metabolites Affect Herbivores: A Study with Pyrrolizidine Alkaloids and Chlorogenic Acid
Source: Front Plant Sci. 2017 May 30;8:903. doi: 10.3389/fpls.2017.00903 (PMC5447715; doi:10.3389/fpls.2017.00903)
Supplement: Supplementary file 1 [file Image_1.PDF]

Supplementary Material

Interactions between plant metabolites affect herbivores: a study with pyrrolizidine alkaloids and chlorogenic acid

Xiaojie Liu*, Klaas Vrieling, Peter G.L. Klinkhamer

*** Correspondence:** Corresponding Author: x.liu.2@biology.leidenuniv.nl

1 Supplementary Figures

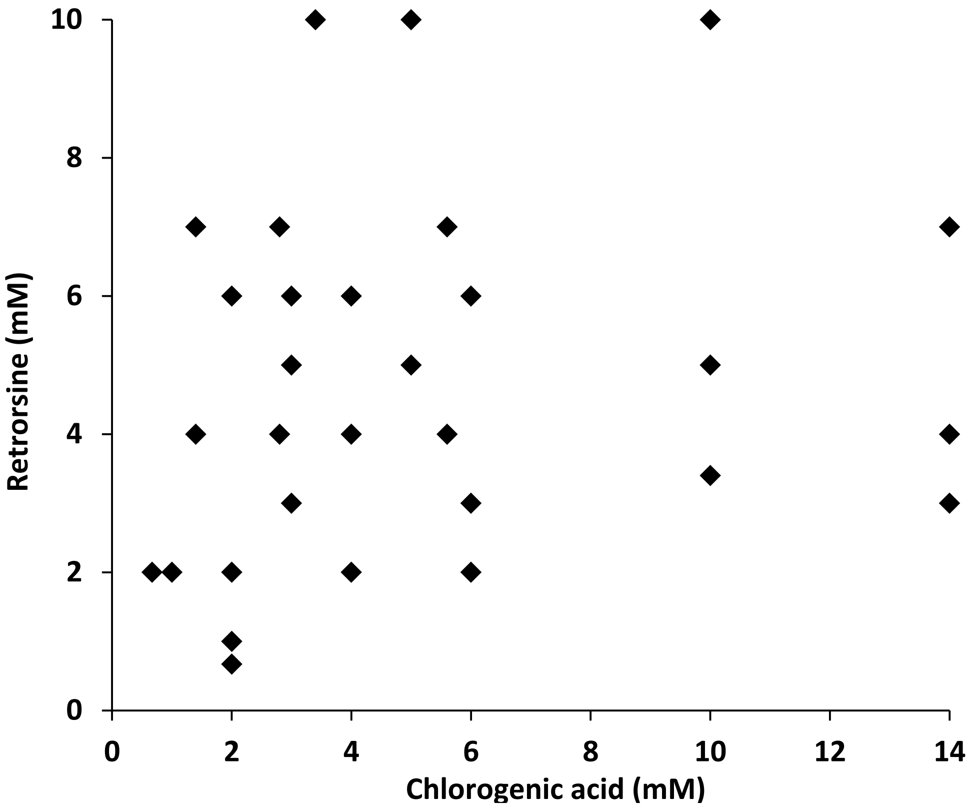

Supplementary Figure 1. Combinations of retrorsine and chlorogenic acid (CGA) tested in the thrips bioassay indicated by black dots.

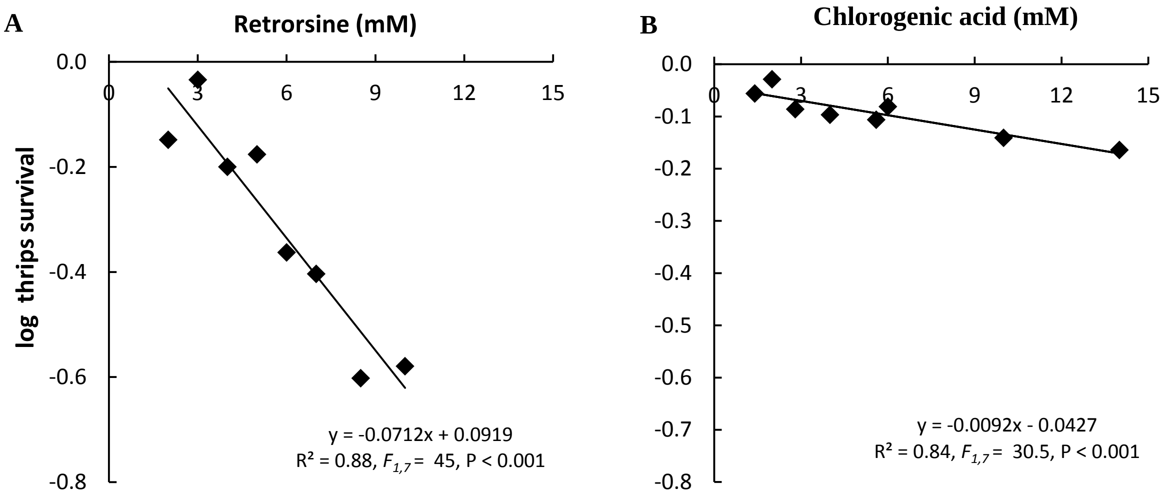

Supplementary Figure 2. Log-transformed survival of 2nd instar *Frankliniella occidentalis* against the concentration of retrorsine **(A)** and chlorogenic acid **(B).**

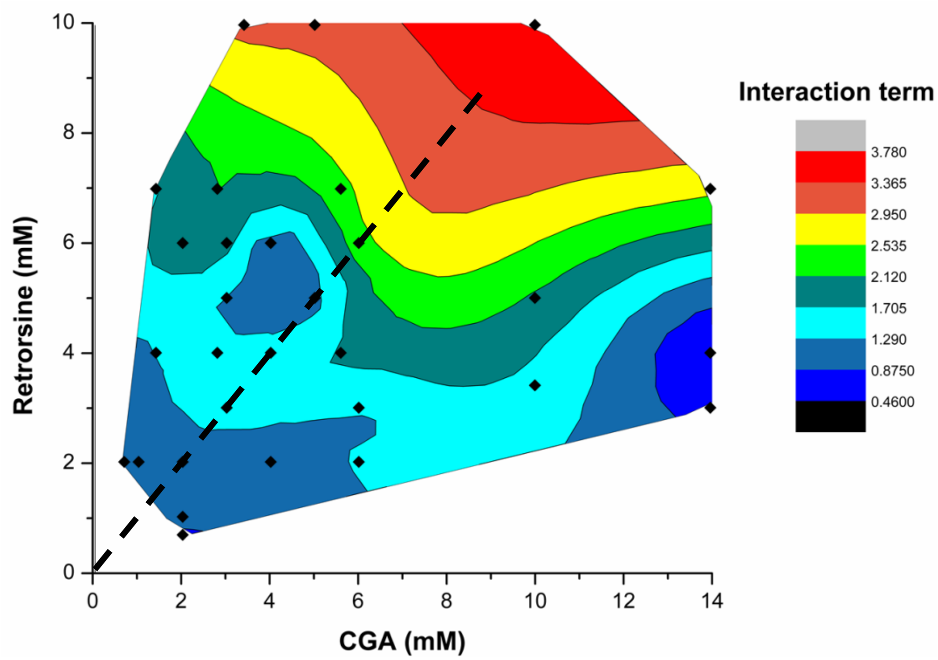

Supplementary Figure 3. Heat-map of the magnitude of the interaction effect (S_{X*Y}) based on thrips (*Frankliniella occidentalis*) survival against retrorsine and chlorogenic acid (CGA) concentrations. Black dots indicate measured values.
